# Using machine learning probabilities to identify effects of COVID-19

**DOI:** 10.1016/j.patter.2023.100889

**Published:** 2023-12-01

**Authors:** Vijendra Ramlall, Undina Gisladottir, Jenna Kefeli, Yutaro Tanaka, Benjamin May, Nicholas Tatonetti

**Affiliations:** 1 Department of Biomedical Informatics, Columbia University, Columbia University Irving Medical Center, New York, NY 10032, USA; 2 Department of Systems Biology, Columbia University, Columbia University Irving Medical Center, New York, NY 10032, USA; 3 Department of Medicine, Columbia University, Columbia University Irving Medical Center, New York, NY 10032, USA; 4 Department of Physiology and Cellular Biophysics, Columbia University, Columbia University Irving Medical Center, New York, NY 10032, USA; 5 Department of Applied Physics and Applied Mathematics, Fu Foundation School of Engineering and Applied Sciences, Columbia University, New York, NY 10027, USA; 6 Herbert Irving Comprehensive Cancer Center, New York Presbyterian/Columbia University Irving Medical Center, New York, NY 10032, USA; 7 Department of Computational Biomedicine, Cedars-Sinai Medical Center, Los Angeles, CA 90069, USA

**Keywords:** COVID-19 effects, clinical informatics, machine learning, algorithm development, survival analysis

## Abstract

Coronavirus disease 2019 (COVID-19), the disease caused by the severe acute respiratory syndrome coronavirus 2 (SARS-CoV-2) virus, has had extensive economic, social, and public health impacts in the United States and around the world. To date, there have been more than 600 million reported infections worldwide with more than 6 million reported deaths. Retrospective analysis, which identified comorbidities, risk factors, and treatments, has underpinned the response. As the situation transitions to an endemic, retrospective analyses using electronic health records will be important to identify the long-term effects of COVID-19. However, these analyses can be complicated by incomplete records, which makes it difficult to differentiate visits where the patient had COVID-19. To address this issue, we trained a random Forest classifier to assign a probability of a patient having been diagnosed with COVID-19 during each visit. Using these probabilities, we found that higher COVID-19 probabilities were associated with a future diagnosis of myocardial infarction, urinary tract infection, acute renal failure, and type 2 diabetes.

## Highlights

• Random Forest classifier was used to predict probability of COVID-19 diagnosis
• Analysis of follow-up identified conditions associated with COVID-19 diagnosis
• Analysis of conditions at different time points show that risk changes over time
• Survival analysis shows COVID-19 is associated with MI risk

## The bigger picture

Understanding the long-term effect of viral infections and disease is vital for treating patients beyond their initial infection—this is particularly important as hundreds of millions of people around the globe have been infected by SARS-CoV-2 and a fraction of those have developed COVID-19, the primary disease caused by SARS-CoV-2. This has been complicated by the novelty of COVID-19 and the limited time span since the first cases and the incompleteness of medical records. In this study, we trained a model to predict whether a patient had been diagnosed with COVID-19 during each visit by using their clinical data. Then we used the output to evaluate more than 1,000 conditions for an association with COVID-19. The methodology we describe in this study can be adapted to understand the effects associated with endemic spikes and can be adapted for any future novel disease.

## Teaser

Studies that aim to identify long-term effects of viral infections and the primary diseases they cause, such as SARS-CoV-2 and COVID-19, rely on medical records; however, their incompleteness is limiting. Ramlall et al. outline a machine learning method to estimate the probability of a patient being diagnosed with COVID-19. The authors use these probabilities to systematically survey 1,039 conditions for associations with higher COVID-19 probability, which identified known conditions associated with COVID-19. The methodology can be adapted to emerging diseases.

## Introduction

The ongoing coronavirus disease 2019 (COVID-19) pandemic, caused by severe acute respiratory syndrome coronavirus 2 (SARS-CoV2) infection of which there have been over 600 million cases around the world, has resulted in more than 6.2 million deaths globally.^1^ In the more than 3 years since the first infection is purported to have occurred,^2^ which prompted the World Health Organization to declare the situation a pandemic between March 2020 and May 2023,^3^ the full impact of SARS-CoV-2 and COVID-19 remains to be seen.

Research has been paramount in responding to the COVID-19 pandemic from identifying patients susceptible to infection and at risk for severe disease^4^^,^^5^^,^^6^ to identifying beneficial treatments^7^^,^^8^^,^^9^ and developing prophylactic measures.^10^^,^^11^^,^^12^ While there have been investigations into the long-term effects of COVID-19,^13^^,^^14^^,^^15^^,^^16^^,^^17^ continual retrospective analyses will be important to identify all the long-term effects and to understand the full scope of the impact of COVID-19.

The long-term effects of viral infections vary greatly. While some viruses, such as certain strains of the seasonal flu and the common cold, have little to no impact on the long-term health of those infected, others can have profound long-lasting effects.^18^^,^^19^ Through long-term analysis, it was determined that varicella zoster, the virus that causes chicken pox, also causes shingles,^20^ a rash accompanied by pain, itching, and tingling, in adults.^21^ Retrospective analyses in patients infected with certain strains of human papilloma virus (HPV) have shown an increased risk of developing anal, cervical,^22^^,^^23^ oropharyngeal, penile, vaginal, and vulvar cancers.^24^ More recently, researchers have identified that Epstein-Barr virus, which causes mononucleosis, also triggers multiple sclerosis,^25^^,^^26^ a demyelinating disease affecting the central nervous system.^27^

Much of the investigations into COVID-19, as well as varicella zoster, HPV, and Epstein-Barr virus infections, have utilized patients’ data sourced from electronic health records (EHRs). While EHRs provide a vast amount of data, such as clinical diagnoses, measurements, and procedures, they were not designed with the intention of being used for research and are incomplete. Research into COVID-19 has been further complicated by the novelty of the disease; the diagnostic *International Classification of Diseases*, 10th edition (ICD-10), code for COVID-19 (U07.1) was not implemented until October 2020.^28^ While the diagnosis code was indicated for COVID-19 as early as April 2020, it was not used for all COVID-19 patients or universally adapted, which has hindered differentiating COVID-19 from non-COVID-19 visits. While EHRs capture SARS-CoV-2 test results, it is not possible to differentiate between an asymptomatic SARS-CoV-2 infection and a COVID-19 case (i.e., SARS-CoV-2 infection with symptoms of the disease) without accompanying diagnosis data.

In this study, we used a random Forest classifier to assign the probability of a patient having been diagnosed with COVID-19 during a given visit (COVID-19 probability). We then used these probabilities to identify downstream conditions associated with a higher probability COVID-19. Specifically, we used a Mann-Whitney *U* test to compare between the distributions of COVID-19 probability of visits that were followed with the diagnosis of a conditions and those that were not at 1 week, 2 weeks, 3 weeks, 4 weeks, 3 months, 6 months, 9 months, and 1 year. Finally, we applied a Cox proportional hazards model adjusting for demographics and frequent clinical phenotypes. We found multiple statistically significant associations between a higher COVID-19 probability and an increased risk for multiple severe outcomes, such as myocardial infarction and acute renal failure.

## Results

**Figure 1:**
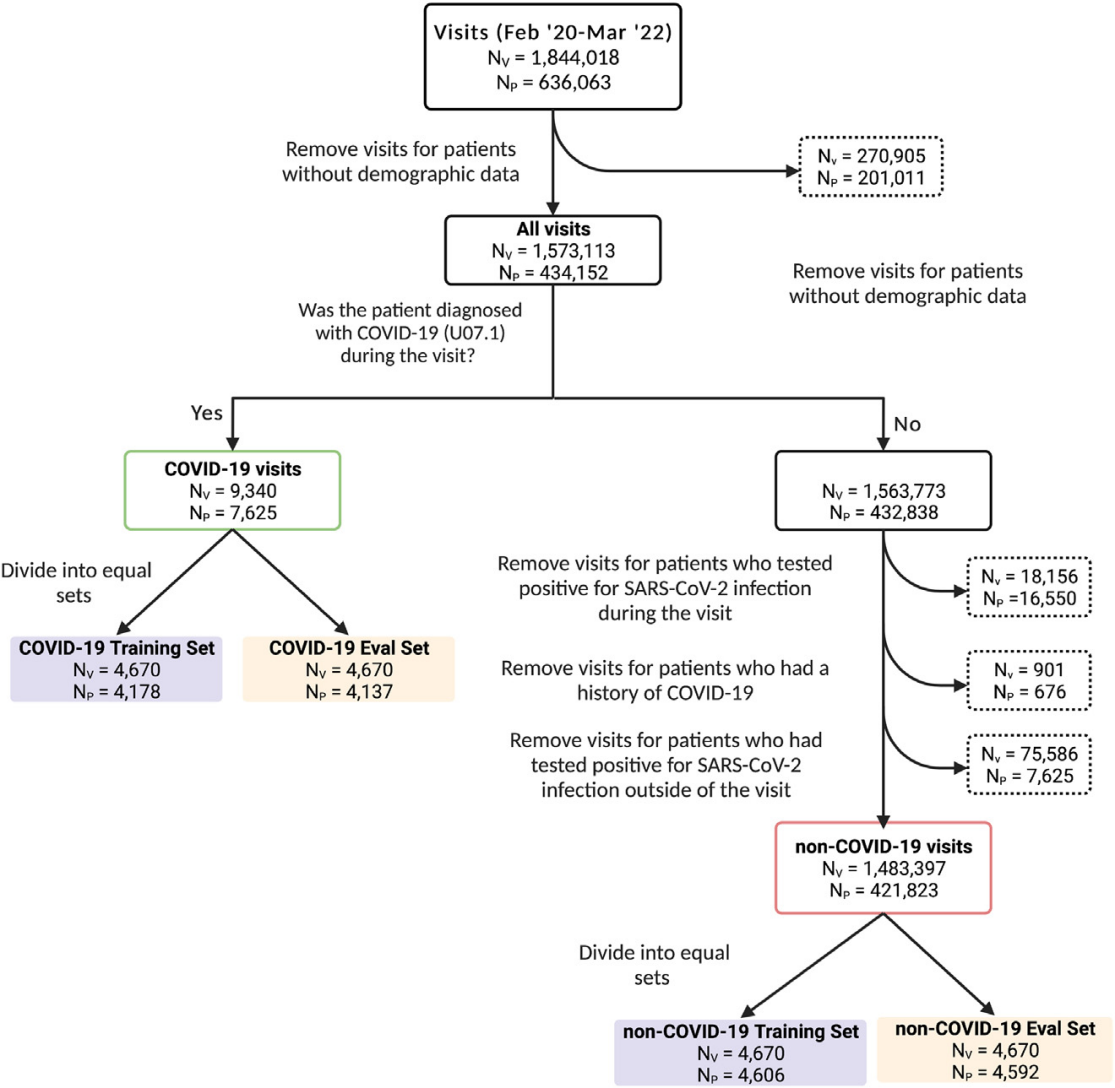
Data processing flowchart Identification of COVID-19 and non-COVID-19 training sets (purple) and evaluation sets (orange). N_V_, the number of visits; N_P_, number of patients in each group. Note: The exclusion criteria used to identify non-COVID-19 visits are not mutually exclusive.

We identified 1,844,018 visits for 636,063 patients who sought treatment at least once between February 1, 2020, and March 31, 2022, at New York Presbyterian/Columbia University Irving Medical Center (NYP/CUIMC). We excluded 270,905 visits for 201,911 patients who did not have any demographic data available in our clinical dataset (Figure 1). From these visits, we identified 9,340 visits where the patient was diagnosed with COVID-19, as evidenced by the presence of the COVID-19 ICD-10 code (COVID-19 visits) (Figure 1). Additionally, we identified 1,483,397 visits where the patient did not test positive for SARS-CoV-2 during that visit, nor had a history of COVID-19 nor previously tested positive for SARS-CoV-2 infection (non-COVID-19 visits) (Figure 1). The set of COVID-19 visit was randomly split into distinct training and evaluation sets, each with 4,670 visits (4,606 and 4,178 patients, respectively) and from the set of non-COVID-19 visits, we randomly identified distinct training and evaluation non-COVID-19 sets, each with 4,670 unique visits (4,592 and 4,137 patients, respectively).

Among all visits, as well as within the COVID-19 and non-COVID-19 training and evaluations sets, more than 50% of the visits were for patients who self-identified as female and more than 85% of the visits were for patients who were at least 19 years old (Table 1). Across all of the groups, more than 35% of the visits were of patients who self-identified as White, more than 15% were of patients who self-identified as Black or African American, and more than 29% were visits of patients who self-identified as Hispanic or of Latino or Spanish origin (Table 1). Across all of the groups, fewer than 5% of visits were of patients who self-identified as American Indian or Alaskan Native, Asian or Native Hawaiian, or Other Pacific Islander (Table 1). Stratification by age, sex, race and ethnicity yielded similar percentages at the patient level (Table S1).

**Table 1:** Demographics of patients visits used for model training, model evaluation and all visits between February 2020 and March 2022

	Model Training Set		Model Evaluation Set		All Visits
	non-COVID-19	COVID-19	non-COVID-19	COVID-19	Feb 2020 - Mar 2022
N(visits)	4,670	4,670	4,670	4,670	1,573,113
N(patients)	4,606	4,178	4,592	4,137	434,152
Age Child (< 13) (% of visits)	365 7.82%	235 5.03%	326 6.98%	242 5.18%	122,140 7.76%
Age Adolescent (≥ 13 and < 19) (% of visits)	146 3.13%	115 2.46%	156 3.34%	115 2.46%	49,232 3.13%
Age Adult (≥ 19 and < 60) (% of visits)	2,375 50.9%	2,159 46.2%	2,324 49.8%	2,099 44.9%	772,606 49.1%
Age Senior (≥ 60) (% of visits)	1,784 38.2%	2,161 46.3%	1,864 39.9%	2,214 47.4%	629,135 40.0%
Self-identified Sex as Female (% of visits)	2756 59.0%	2412 51.6%	2885.0 61.8%	2403.0 51.5%	941558.0 59.9%
Self-identified as American Indian or Alaskan Native (% of visits)	14 0.300%	18 0.385%	11 0.236%	< 10 < 0.214%	3,994 0.254%
Self-identified as Asian (% of visits)	108 2.31%	124 2.66%	120 2.57%	116 2.48%	39,091 2.48%
Self-identified as Black or African American (% of visits)	728 15.6%	803 17.2%	702 15.0%	811 17.4%	245,104 15.6%
Self-identified as Native Hawaiian or Other Pacific Islander (% of visits)	< 10 < 0.214%	10 0.214%	< 10 < 0.214%	< 10 < 0.214%	1,520 0.0966%
Self-identified as White (% of visits)	1,900 40.7%	1,645 35.2%	1,974 42.3%	1,669 35.7%	643,848 40.9%
Self-identified as Hispanic or of Latino or Spanish Origin (% of visits)	1,433 30.7%	1,873 40.1%	1,367 29.3%	1,782 38.2%	473,501 30.1%

Among all visits, the largest fraction of visits (5.17%) began in March 2021 (Table S2). The largest fraction of visits in the COVID-19 training and evaluation sets began in April 2020 (18.29% and 17.99%, respectively), while the smallest fraction of all visits began in April 2020 (1.64%) (Table S2). The fraction of visits in the non-COVID-19 training and evaluation sets that began in each month were similar to the faction of all visits that began in each month (Table S2). Among all visits, the four most frequently used diagnosis codes were for “supervision of normal pregnancy” (2.38%), “transplanted organ and tissue status" (2.26%), “other symptoms and signs involving the circulatory and respiratory system” (2.18%), and “essential (primary) hypertension” (2.06%) (Table S3). These diagnosis codes were similarly the most frequently used among non-COVID-19 visits in the training and evaluation sets (Table S3). Among the COVID-19 visits in the training and evaluation sets, “diagnosis of other symptoms and signs involving the circulatory and respiratory system” (20.75% and 19.21%, respectively), “encounter for screening for malignant neoplasms” (19.46% and 19.08%, respectively), “essential (primary) hypertension” (8.84% and 9.27%, respectively), and “transplanted organ and tissue status” (8.22% and 8.78%, respectively) were the most frequently diagnosed (Table S3).

**Figure 2:**
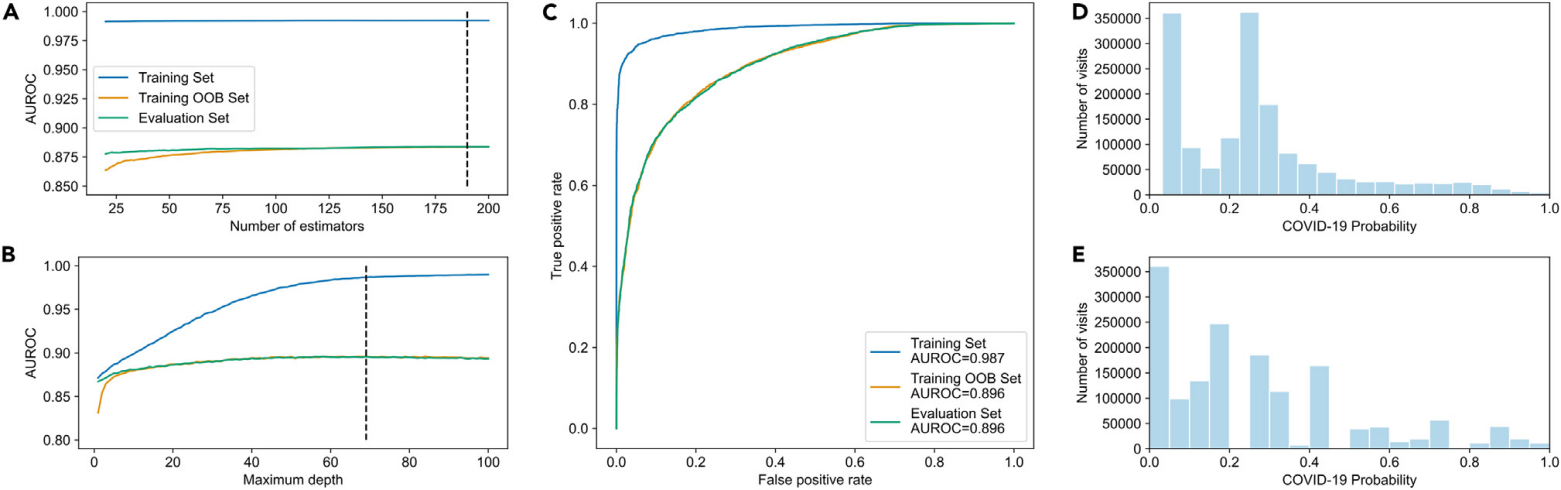
Model performance optimization (A) AUROC in training set, training set using OOB estimates, and evaluation set plotted against number of estimators (dashed line indicates maximum AUROC in evaluation set, n_estimators = 190). (B) AUROC in training set, training set using OOB estimates, and evaluation set plotted against maximum depth (dashed line indicates maximum AUROC in evaluation set, max_depth = 69). (C–E) (C) ROC curves of training set, training set using OOB estimates, and evaluation set. Distribution of COVID-19 probabilities in all visits prior to calibration (D) and after calibration (E).

We collected demographic data of the patient in each visit (date of birth, self-identified sex, self-identified race[s] and self-identified ethnicity), temporal data (during which month the visit started) and visit specific diagnosis data. We used a random Forest classifier to predict whether or not a patient was diagnosed with COVID-19 during their visit using demographic, temporal, and visit-specific clinical diagnoses, which were identified at the category level. Instead of the binary classification from the random Forest classifier (patient diagnosed with COVID-19 during the visit or patient not diagnosed with COVID-19 during the visit), we treated the fraction of trees that classified the visit as one where the patient was diagnosed with COVID-19 as the probability of that scenario. An initial random Forest classifier of 200 estimators was fit using the COVID-19 and non-COVID-19 training sets using bootstrapped sampling and out-of-bag (OOB) sampling (training area under the receiver operating characteristic curve [AUROC] = 0.992, training OOB AUROC = 0.884, evaluation AUROC = 0.884) (Figure S1A). To optimize model performance, we monitored the AUROC of the training set, the training set using OOB estimates and the evaluation set while increasing the number of estimators from 20 to 200 and achieved a maximum AUROC in the evaluation set with 190 estimators (training set AUROC = 0.992, training set OOB AUROC = 0.884, evaluation set AUROC = 0.884) (Figure 2A). We further optimized model performance by monitoring the AUROC while increasing the maximum depth of the model from 1 to 100 with 190 estimators and achieved a maximum AUROC in the evaluation set with a depth of 69 (training set AUROC = 0.9867, training set OOB AUROC = 0.8957, evaluation set AUROC = 0.858) (Figure 2B). The optimized model trained with 190 estimators with a maximum depth of 69 was fit to the data representing all 1.57 million visits (Figure 2C). The predicted probabilities from the random Forest classifier were aligned with the true probability of a COVID-19 diagnosis using an isotonic calibration (Figures 2D, 2E, and S1B–S1E).

**Figure 3:**
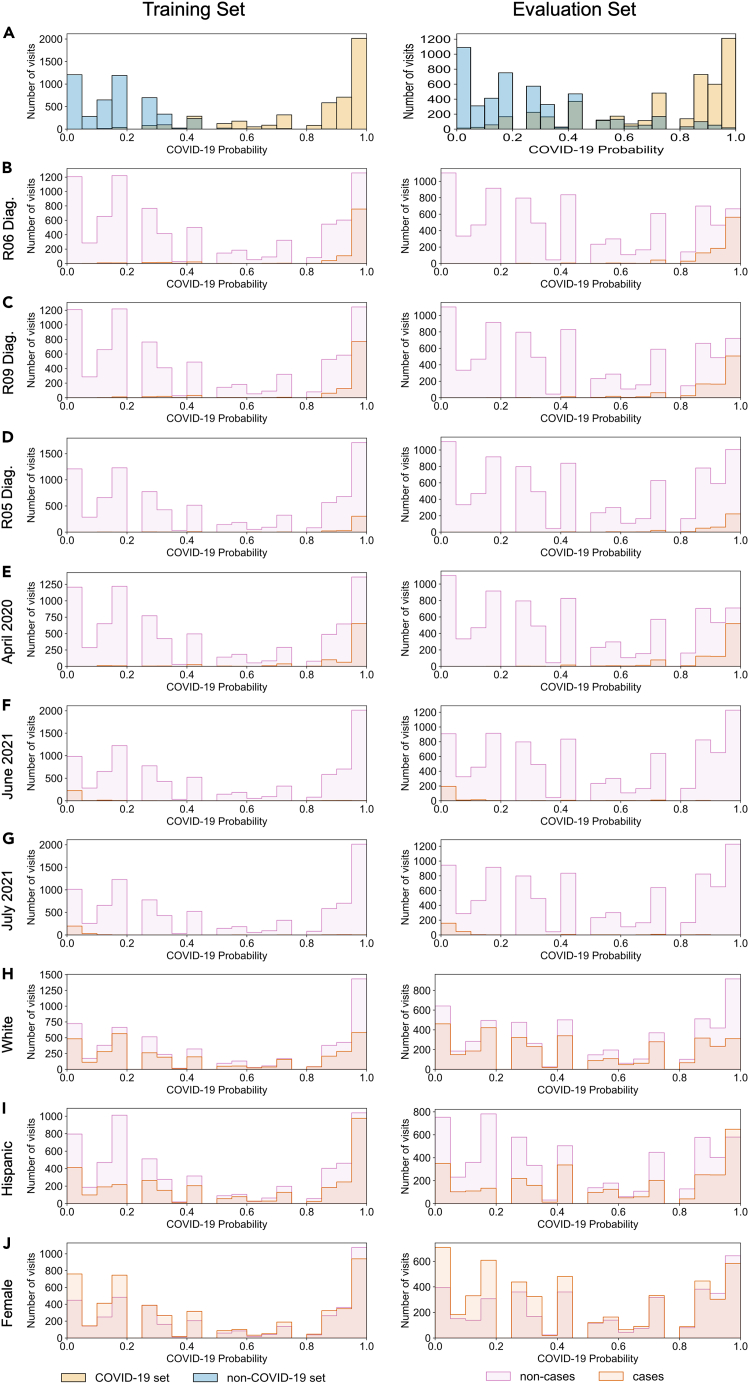
Analysis of important features (A) Distribution of COVID-19 probability in COVID-19 and non-COVID-19 training (left) and evaluation sets (rights). Distribution of cases (red) and non-cases (purple) for important diagnoses (B–D), temporal (E–G), and demographic (H–J) features for training and evaluation sets. Note: R05, cough, R06, abnormalities of breathing; R09, other symptoms and signs involving the circulatory and respiratory system diagnosis noted during the visit.

We evaluated the features utilized in the final model using the Gini importance (Tables 2 and Table S4). Diagnosis of abnormalities of breathing (R06), other symptoms and signs involving the circulatory and respiratory system (R09), and cough (R05) during the visit had the highest importance of the conditions included in the final model (Table 2). The distribution of the COVID-19 probabilities of the visits where these diagnoses were noted were shifted toward higher COVID-19 probability compared with visits where these diagnoses were not noted in both the training and evaluation sets (Wasserstein distance [WD] = 0.479, 0.474, and 0.464, respectively in the training set) (Figures 3B–3D and Table 2). Visits that started in April 2020, June 2021, and July 2021 were the temporal features with the highest importance in the final model (Table 2). The distribution of the COVID-19 probabilities of visits that started in April 2020 were shifted toward higher COVID-19 probabilities compared with those that did not start in April 2020 (WD = 0.461 in the training set) (Figure 3E and Table 2). Conversely, the distributions of the COVID-19 probabilities of visits that started in June 2021 and July 2021 were shifted toward lower COVID-19 probabilities compared with those that started at other times (WD = −0.430 and −0.426, respectively, in the training set) (Figures 3F and 3G and Table `2). Patients self-identifying as White, of Hispanic or Latino or Spanish origin, and female were the demographic features with the highest importance in the final model (Table 2). The distributions of COVID-19 probabilities of visits in which the patient self-identified as White or female were shifted toward lower COVID-19 probabilities compared with those where the patient did not (WD = −0.0615, −0.0772, respectively, in the training set) (Figures 3H and 3J and Table 2). The distribution of COVID-19 probabilities of visits in which the patient self-identified as of Hispanic or Latino or Spanish origin were shifted toward higher COVID-19 probabilities compared with those where the patient did not (WD = 0.103) (Figure 3I and Table 2).

**Table 2:** Importance for the top 20 important features and Wasserstein distance between distribution where the feature is observed and the feature is not observed. Negative Wasserstein distance indicates that the average COVID-19 probability in the set of visits where the feature was observed is less than the average of the set where the feature was not observed

Feature	Importance	Wasserstein Distance		
		Training Set	Evaluation Set	All Visits
Abnormalities of breathing diagnosis noted during visit (R06)	0.0650	0.479	0.487	0.577
Other symptoms and signs involving the circulatory and respiratory system diagnosis noted during visit (R09)	0.0628	0.474	0.463	0.546
Visit started in April 2020	0.0543	0.461	0.461	0.492
Cough diagnosis noted during visit (R05)	0.0259	0.464	0.451	0.582
Viral pneumonia, not elsewhere classified diagnosis noted during visit (J12)	0.0236	0.507	0.488	0.704
Encounter for other special examination without complaint, suspected or reported diagnosis noted during visit (Z01)	0.0234	0.333	0.351	0.510
Transplanted organ and tissue status diagnosis noted during visit (Z94)	0.0229	0.340	0.360	0.496
Fever of other and unknown origin diagnosis noted during visit (R50)	0.0195	0.460	0.443	0.578
Respiratory failure, not elsewhere classified diagnosis noted during visit (J96)	0.0176	0.507	0.498	0.656
Self-identified as White	0.0148	−0.0615	−0.0647	−0.0104
Visit started in June 2021	0.0148	−0.430	−0.413	−0.235
Self-identified as of Hispanic or Latino or Spanish Origin	0.0141	0.103	0.101	0.0419
Self-identified Sex as Female	0.0141	−0.0772	−0.0867	−0.0269
Visit started in July 2021	0.0130	−0.426	−0.411	−0.224
Visit started in August 2021	0.0126	−0.386	−0.420	−0.205
Visit started in September 2021	0.0117	−0.388	−0.378	−0.221
Visit started in February 2020	0.0115	−0.440	−0.366	−0.144
Visit started in October 2021	0.0112	−0.397	−0.415	−0.223
Type 2 diabetes mellitus diagnosis noted during visit (E11)	0.0106	0.419	0.420	0.513
Acute kidney failure diagnosis noted during visit (N17)	0.0104	0.483	0.480	0.599

**Figure 4:**
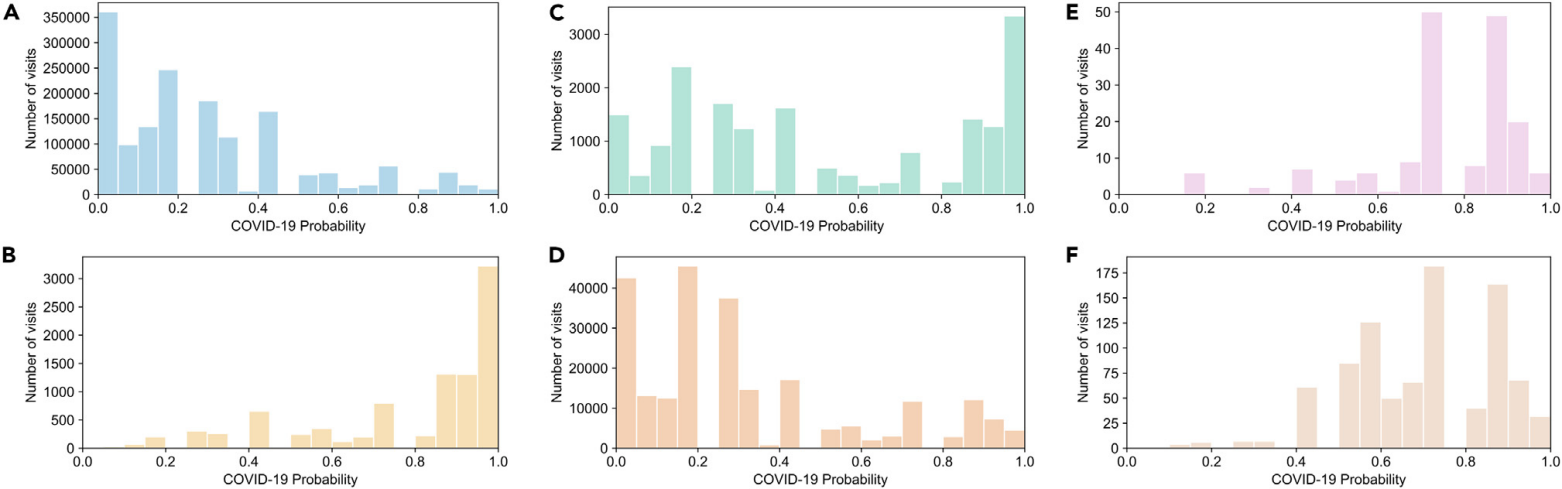
Distribution of COVID-19 probability for visits different patient groups (A) Distribution of COVID-19 probability for all visits. Distribution of visits where patients were diagnosed with COVID-19 (B), tested positive for SARS-CoV-2 infection (C), tested negative for SARS-CoV-2 infection (D), where clinical diagnosis note indicated that COVID-19 was ruled out, (E) and visits where the patient had a history of COVID-19 (F).

We further evaluated the model by evaluating the distributions of COVID-19 probabilities for visits within inclusion and exclusion criteria for the training and evaluation sets (Figure 4). Compared with the distribution of COVID-19 probabilities for all of the visits between February 2020 and March 2022 (Figure 4A), the distribution of COVID-19 probabilities for visits where the patient was diagnosed with COVID-19 (N = 9,340) during the visits were shifted toward higher probabilities (WD = 0.522) (Figure 4B). The distribution of COVID-19 probabilities of visits where the patient tested positive for SARS-CoV-2 infection (N = 18,156) was bimodal with a shift toward higher COVID-19 probabilities (WD = 0.254) (Figure 4C). The distribution of COVID-19 probabilities of visits where the patient tested negative for SARS-CoV-2 infection (N = 238,438) was marginally shifted to higher COVID-19 probabilities (WD = 0.067) (Figure 4D). The distribution of COVID-19 probabilities of visits where clinical diagnosis notes indicated that the patient did not have COVID-19 (N = 168) was shifted to higher COVID-19 probabilities (WD = 0.492) (Figure 4E). The distribution of COVID-19 probabilities of visits where the patient had a noted history of COVID-19 (N = 899) was shifted to higher COVID-19 probabilities (WD = 0.431) (Figure 4F).

**Figure 5:**
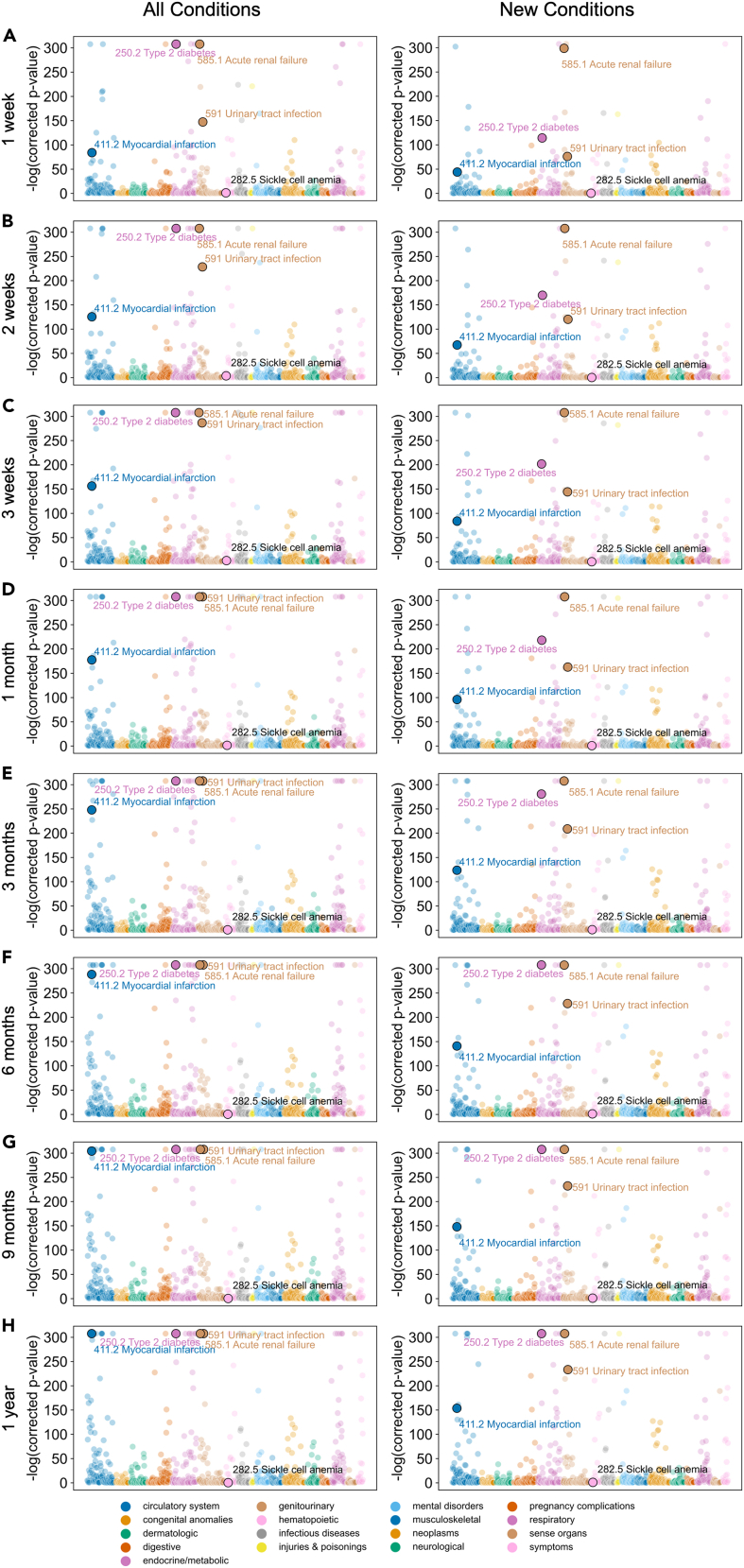
Statistical testing of conditions associated with COVID-19 Manhattan plots of –log10(corrected p value) from MWU test between distributions of COVID-19 probabilities of cases and non-cases for each phenotype (colored by family) within (A) 1 week, (B) 2 weeks, (C) 3 weeks, (D) 1 month, (E) 3 months, (F) 6 months, (G) 9 months, and (H) 1 year for all instances of conditions (left) and only new onset of the conditions (right).

To identify conditions that are associated with a history of COVID-19, we identified visits where the patient returned to the hospital within 7 days, 14 days, 21 days, 28 days, 3 months, 6 months, 9 months, and 12 months by comparing the distributions of COVID-19 probabilities of visits preceding those in which the patient returned with the censored time and a particular condition was observed in the follow-ups and those where the condition was not. We used a Mann-Whitney *U* (MWU) test to compare the distribution of COVID-19 probabilities that preceded the occurrence of the condition to the distribution of COVID-19 probabilities that did not preceded the occurrence of the condition for each condition for all instance of the condition occurring (Figure 5 left and Table S5) and only if the condition was newly diagnosed (Figure 5 right and Table S5). Among the conditions reviewed, the distribution of COVID-19 probability preceding myocardial infarction (MI) was significantly different from the distribution of COVID-19 probability not preceding MI infarction in analysis of all instances of MI and of new diagnoses in all time periods (MWU = 1.35E8; false discovery rate [FDR]-corrected p < 2.3E−308; MWU = 5.69E7 FDR-corrected p < 2.13E−154, respectively, within 1 year) (Figure 5 and Tables 3 and S5). We observed a similar difference in analyses of all instances and only new diagnoses of urinary tract infection (UTI) (MWU = 2.58E8; FDR-corrected p < 5.13E−234; MWU = 8.83E7; FDR-corrected p *=* 5.13E−234 within 1 year), acute renal failure (MWU = 1.25E8; FDR-corrected p < 2.225E−308; MWU = 4.66E7; FDR-corrected p < 2.225E−308 within 1 year), and type 2 diabetes (T2D) (MWU = 3.29E8; FDR-corrected p < 2.225E−308; MWU = 4.93E7; FDR-corrected p < 2.225E−308 within 1 year) (Figure 5 and Tables 3 and S5). We did not observe a similar difference in the analysis of either all instances nor only new diagnoses of sickle cell anemia (MWU = 7.13E8; FDR-corrected p = 0.494; MWU = 5.40E7; FDR-correct p = 0.108 within 1 year) (Figure 5 and Tables 3 and S5).

**Figure 6:**
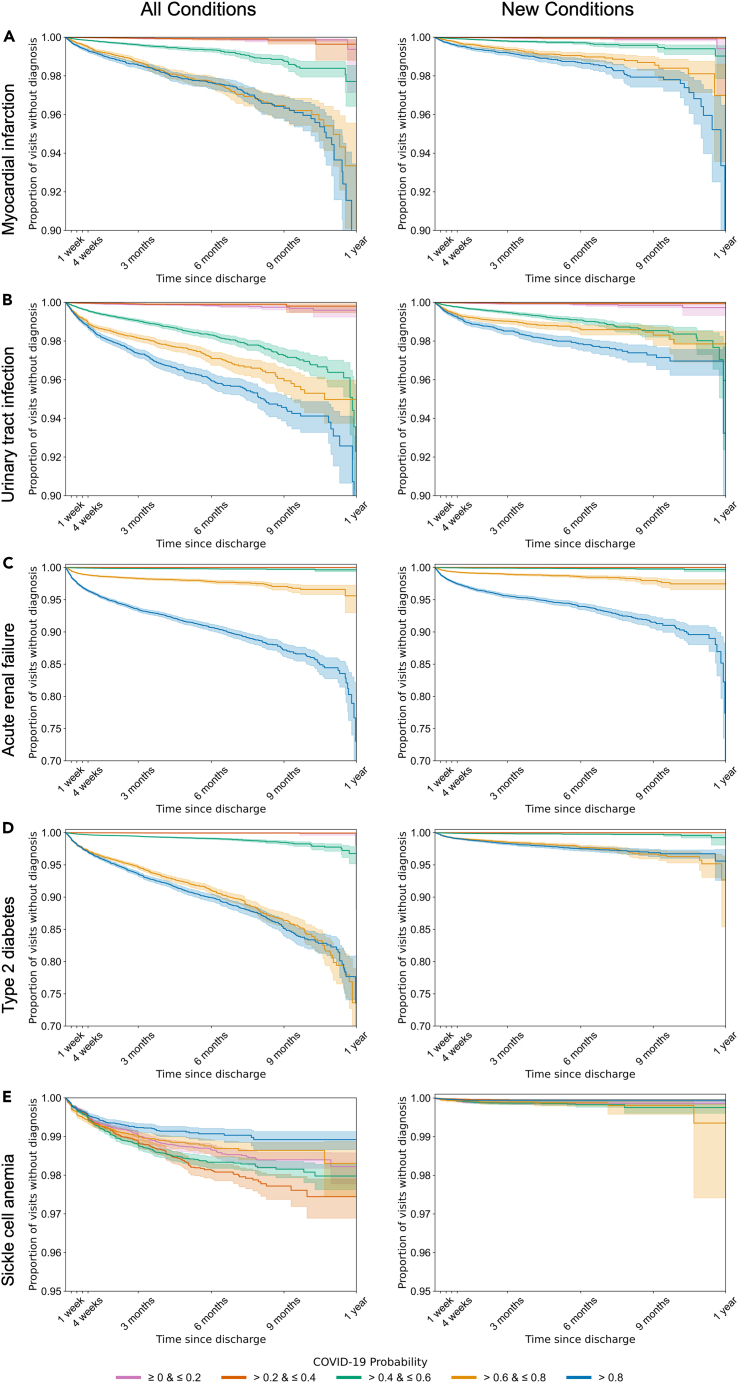
Kaplan-Meier curves for select conditions Kaplan-Meier curves for (A) MI, (B) UTI, (C) acute renal failure, (D) T2D, and (E) sickle cell anemia stratified by COVID-19 probability quintile within 1 year for all instances of the conditions (left) and only new onset of the conditions (right). Shaded regions indicate the 95% confidence interval for the corresponding quintile.

To further investigate the association between COVID-19 probability and the onset of conditions, we calculated the hazard ratio using a Cox proportional hazards model for COVID-19 probability using (a) all visits regardless of the patient’s previous clinical history and (b) visits for patients who did not have a previous history of the condition (Table S6). Increasing COVID-19 probability in the preceding visit was associated with an increased risk of diagnosis of MI when patients who had previous diagnosis of MI were included and excluded from the analysis (hazard ratio [HR], 70.0; 95% confidence interval [VI], 55.2–88.8; p = 8.61E−270 and HR, 88.6; 95% CI, 62.4–126; p = 7.06E−139, respectively) (Table 3). A similar association was observed within 1 year in both analyses for UTI (HR, 49.4; 95% CI, 41.7–58.6; p < 2.225E−308 and HR, 53.3; 95% CI, 41.2–69.0; p = 4.41E−200, respectively), in both analyses of acute renal failure (HR, 8.93E3; 95% CI, 6.72E3–1.19E4; p < 2.225E−308 and HR, 1.42E4; 95% CI, 9.48E3–2.12E4; p < 2.225E−308, respectively) and in both analyses of T2D (HR, 293; 95% CI, 253–340; p < 2.225E−308 and HR, 325; 95% CI, 235–451; p = 7.91E−263, respectively) (Figure 6 and Table 3). COVID-19 probability in the preceding visiting was not significantly associated with an altered risk of diagnosis of sickle cell anemia when patients who had a previous diagnosis of sickle cell anemia were included in the analysis (HR, 0.900; 95% CI, 0.777–1.04; p = 0.164) (Figure 6 and Table 3). However COVID-19 probability in the preceding visit was significantly associated with a lower risk of sickle cell anemia when patients who had a previous history were excluded from the analysis (HR, 0.557; 95% CI, 0.328–0.946; p = 3.04E−02) (Figure 6 and Table 3).

**Table 3:** Results of Mann-Whitney U test, univariate Cox proportional hazards ratio and multivariate Cox proportional hazards ratio of COVID-19 probability within 1 year for select conditions

		Mann-Whitney U Test (Test statistic, *p* value, FDR corrected *p* value	Cox Proportional Hazards Univariate Fit (Hazards ratio, 95% CI, *p* value)	Cox Proportional Hazards Multivariate Fit (Hazards ratio, 95% CI, *p* value)
ALL CONDITIONS	Myocardial infarction (411.2)	1.35E+08 1.34E-299 < 2.23E-308	70.0 (55.2, 88.8) 8.61E-270	68.7 (53.4, 88.3) 6.42E-239
	Urinary tract infection (591)	2.58E+08 1.34E-299 < 2.23E-308	49.4 (41.7, 58.6) < 2.23E-308	41.3 (34.6, 49.3) <2.23E-308
	Acute renal failure (585.1)	1.25E+08 1.34E-299 < 2.23E-308	8.93E+03 (6.72E+03, 1.19E+04) < 2.23E-308	8.31E+03 (6.17E+03, 1.12E+04) <2.23E-308
	Type 2 diabetes (250.2)	3.29E+08 1.34E-299 < 2.23E-308	293 (253, 340) < 2.23E-308	174 (149, 203) <2.23E-308
	Sickle cell anemia (282.5)	7.13E+08 0.490 0.494	0.900 (0.777, 1.04) 0.164	0.627 (0.537, 0.732) 3.70E-09
NEW CONDITIONS	Myocardial infarction (411.2)	5.69E+07 7.11E-156 2.13E-154	88.6 (62.4, 126) 7.06E-139	109 (76.3, 157) 1.22E-143
	Urinary tract infection (591)	8.83E+07 1.01E-235 5.13E-234	53.3 (41.2, 69.0) 4.41E-200	64.3 (49.3, 84.0) 5.76E-206
	Acute renal failure (585.1)	4.66E+07 3.87E-303 < 2.23E-308	1.42E+04 (9.48E+03, 2.12E+04) <2.23E-308	1.69E+04 (1.11E+04, 2.57E+04) <2.23E-308
	Type 2 diabetes (250.2)	4.93E+07 3.87E-303 < 2.23E-308	325 (235, 451) 7.91E-263	386 (278, 536) 7.91E-276
	Sickle cell anemia (282.5)	5.40E+07 0.071 0.108	0.557 (0.328, 0.946) 3.04E-02	1.26 (0.746, 2.14) 0.385

To further understand the association identified in our study between COVID-19 probability and diagnosis of MI, UTI, acute renal failure, T2D, and sickle cell anemia, we used a multivariate Cox proportional hazards model that incorporated age, sex, race, ethnicity and previous clinical history for the 30 most frequently observed diagnoses in the analysis (Table S7). In an analysis of all patients who had a follow-up visit within 1 year, we observed a similar situation in our multivariate modeling as in the univariate Cox proportional hazards modeling. There was a significant association between higher COVID-19 probabilities in the preceding visit and an increased risk of diagnosis of MI (HR, 68.7; 95% CI, 53.4–88.3; p = 6.42E−239), UTI (HR, 41.3; 95% CI, 34.6–49.3; p < 2.23E−308), acute renal failure (HR, 8.31E3; 95% CI, 6.17E4–1.12E4; p < 2.23E−308) and T2D (HR, 174; 95% CI, 149–203; p < 2.23E−308) (Table 3). There was a significant association between higher COVID-19 probabilities in the preceding visit and a decreased risk of diagnosis of sickle cell anemia (HR, 0.627; 95% CI, 0.537–0.732; p = 3.70E−9) (Table 3). When focusing on only newly diagnosed conditions, our results in the multivariate modeling were similar to those in the univariate modeling. There was a significant association between higher COVID-19 probabilities in the preceding visit and an increased risk of the first diagnosis of MI (HR, 109; 95% CI, 76.3–157; p = 1.22E−143), UTI (HR, 64.3; 95% CI, 49.3–84.0; p = 5.76E−206), acute renal failure (HR, 1.69E4; 95% CI, 1.11E4–2.57E4; p < 2.23E−308) and T2D (HR, 386; 95% CI, 278–536; p = 7.91E−276) (Table 3). However, there was not a significant association between COVID-19 probability in the preceding visit and the new diagnosis of sickle cell anemia when accounting for demographics and previous clinical history in the modeling (HR, 1.26; 95% CI, 0.746–2.14; p = 0.385) (Table 3).

Finally, we wanted to understand how a higher or lower COVID-19 probability influenced the incidence of conditions over time. Since COVID-19 probability was treated as a continuous variable in our analysis, we segmented the probabilities into quintiles and generated Kaplan-Meier curves of each COVID-19 probability quintile for MI, UTI, acute renal failure, T2D, and sickle cell anemia when including patients who had a previous diagnosis (Figure 6, left) and when focusing only on new diagnoses of the condition (Figure 6, right). The Kaplan-Meier curves that focused on all instances of MI (Figure 6A, left) and new diagnoses of MI (Figure 6A ,right) showed three distinct groups: (i) COVID-19 probability greater than 0.6, (ii) COVID-19 probability greater than 0.4 and less than or equal to 0.6, and (iii) COVID-19 probability less than or equal to 0.4, with a higher incidence observed in the sets of higher COVID-19 probability (Figure 6A). Similarly, the Kaplan-Meier curves that focused all instances of UTI (Figure 6B, left) and new diagnoses of UTI (Figure 6B, right) showed a similar grouping of the two lowest quintiles with low incidence rate. The three remaining quintiles showed distinct patterns with a positive correlation between the COVID-19 probability and the incidence of UTI and a convergence near 1 year since discharge; the incidence rate among the three highest quintiles when analyzing all instances of UTI was approximately twice that when only analyzing new onset. The Kaplan-Meier curves that focused on all instances of acute renal failure (Figure 6C, left) and new diagnoses of acute renal failure (Figure 6C, right) showed a group of the three lowest quintiles with the lowest incidence rate and distinct patterns in the remaining two quintiles with a positive correlation between the COVID-19 probability and the incidence rate. The Kaplan-Meier curves that focus on all instances of T2D (Figure 6D, left) and new diagnoses of T2D (Figure 6D, right) showed a grouping similar to that observed for MI; however, the incidence rate for all instance of T2D was approximately five times that the incidence of new diagnoses of T2D. Finally, the Kaplan-Meier curves for all instance of sickle cell anemia (Figure 6E, left) and new diagnoses of sickle cell anemia (Figure 6E, right) showed a single conglomerate group with no discernible association between COVID-19 probability and incidence rate, though the incidence rate of all instances of sickle cell anemia was two to six times the rate of incidence of new diagnose of sickle cell anemia.

## Discussion

In this study, we collected demographic, temporal and clinical data from patients who sought treatment at NYP/CUIMC between February 2020 and March 2022 to develop an algorithm to identify conditions that are associated with COVID-19. The 26-month period from which our data is sourced encompasses the height of the first wave of the COVID-19 pandemic (Spring 2020) when New York City was an epicenter in the United States as well as the subsequent Delta and Omicron waves.^29^ Additionally, our data encompass periods with low case counts, such as summer 2020, as well as the period after the development of treatments for COVID-19 and prophylactics for SARS-CoV-2 infection.

Using data for patients diagnosed with COVID-19 and non-COVID-19 patients, we trained and optimized a random Forest classifier and then applied our trained model to estimate the probability of a patient having been diagnosed with COVID-19 during each of more than 1.57 million visits. While the random Forest classifier overfits on the data (based on the high AUROC observed in the training set), it performed similarly in the training set using OOB estimates and the evaluation set.

When evaluating our model, the most important features represented previously identified differences between demographic groups, such as those who self-identify as Hispanic or Latino or of Spanish origin or Black or African American^30^^,^^31^ (Table 2 and Figure 3). Important temporal features represented periods of case count extremes in New York City, such as spring 2020 and summer 2021,^27^ and correlated with the positivity rate, testing capabilities and hospitalization rates in New York City, indicating that it may be acting as a proxy for them in our model (Table 2 and Figures 3 and S2). Important clinical diagnoses were reflective of known symptoms of COVID-19,^32^ such as abnormalities of breathing (R06), other symptoms and signs involving the circulatory and respiratory system (R09), and cough (R05) (Table 2 and Figure 3). Using these visit specific probabilities, we identified conditions that developed within different time periods after the visit.

Cox proportional hazards model indicated that higher COVID-19 probability in the preceding visit was associated with an increased risk of MI, UTI, acute renal failure, and T2D within 1 year. Our top results recapitulate COVID-19-associated conditions found within other studies. Our result for MI is consistent with those of researchers who identified a higher risk of heart attack and ischemic stroke in COVID-19 patients using self-controlled case series.^14^ Results from a retrospective observational study of patients in early 2020 observed that severe COVID-19 disease is associated with acute kidney injury.^16^ Other researchers have identified an increased risk of T2D in patients who had been infected with SARS-CoV-2 compared with patients who had not and compared with a historical control.^17^ While it is difficult to identify a control in studies such as ours, we used sickle cell anemia, which is a genetic disease usually screened for at birth, since we did not expect there to be any relationship between sickle cell anemia diagnosis and COVID-19. This finding is supported by our survival analysis results which show that the COVID-19 HR is not significant in predicting the diagnosis of sickle cell anemia when controlling for demographics and previous clinical history. While we report HRs for each condition, we caution that they should not be evaluated in an absolute context since COVID-19 probability was indicated on a 0 to 1 scale and the estimated HRs are limited by the study population.

The Kaplan-Meier curves for MI, UTI, acute renal failure, and T2D show different patterns when COVID-19 probability is generalized into quintiles (Figure 6). While the two lowest quintiles are generally reflective of low or no incidence of disease, the relative clustering of the three remaining quintiles vary. It is our hypothesis that the COVID-19 probability is correlated with disease severity and the clustering observed may be due to the importance of the comorbidities in the random Forest classifier, which may correlate with the patient’s underlying risk of developing the disease.

While this study shows that demographic, temporal, and clinical data can be utilized to predict the probability of a patient having been diagnosed with COVID-19 during their visit, the model and the important features are specific to NYP/CUIMC. An implementation of this model elsewhere is expected to identify important site-specific temporal features (e.g., periods of extreme case counts varied between New York City and London) and demographic variables depending on the patient populations seeking treatment at those sites. However, it would be expected to identify similar clinical variables that are representative of known symptoms or comorbidities associated with COVID-19. Random Forest algorithms are dependent on the quality of the input data, so the completeness and accuracy of the visit specific data is important for estimating COVID-19 probability with high confidence.

While our results concur with other studies, they are not without their biases; this study relied on patients who sought treatment at NYP and was unable to incorporate data from patients who may have also sought outside treatment due to the nature of primary care in the United States. The demographics of the patients who sought treatment at NYP/CUIMC are not representative of the New York City’s population; based on location alone, nuances in the Hispanic population to be more easily identified than those among the Asian population due to representation within our dataset. Additionally, our study relies on ICD-10 diagnostic codes, both in identifying COVID-19 cases and controls and identifying potential long-term conditions to develop a streamlined approach that can be easily adapted to different organizations and different diseases.

The analysis of sickle cell anemia highlights an inherent limitation of working with clinical data and when looking at low incident events. Counterintuitively, multivariate Cox proportional hazards modeling using all instances of sickle cell anemia, increasing COVID-19 probability was associated with increased risk of a future diagnosis of sickle cell anemia. We are confident in our analyses for the other conditions discussed due to the relatively low significance of the COVID-19 probability HR in the Cox proportional hazards model and the Kaplan-Meier curves did not show a discernible correlation between COVID-19 probability and incidence of sickle cell anemia. It is our hypothesis that this may be due to increased interactions with the hospital for patients with sickle cell anemia for ongoing and emergency care. Additionally, the change in significance of COVID-19 probability between the univariate and multivariate Cox proportional hazards modeling for new diagnoses of sickle cell anemia show the importance of including demographic and clinical features since there are known racial biases in the diagnosis of disease.

In identifying effects of COVID-19, we are limited by the novelty of the disease itself; other effects may take years or even decades to develop and our data are limited to conditions occurring up to 1 year after the COVID-19 visit. Additionally, disease pathogenesis may differ between age and racial or comorbidity groups, which our dataset may not have captured. To that end, our multivariate Cox proportional hazards modeling incorporated demographic features, such as age, and the COVID-19 HR was similar to the HR in the univariate model.

Our study demonstrated a new method to conduct retrospective analyses for identifying the effects of COVID-19. By implementing a model trained on clinical data at the visit level and using the output from a random Forest classifier as a probability instead of a binary outcome, we mitigated the need to definitively distinguish cases. Additionally, the results from our study can be used to direct further investigations into the effects of COVID-19. As COVID-19 transitions from a pandemic to an endemic, our method can be utilized to understand potential pathophysiological differences in symptoms associated with COVID-19 spikes. Moreover, as this method was designed using concurrent clinical data, it can be adapted to other novel or emerging diseases.

## Experimental procedures

### Resource availability

#### Lead contact

Request for further information should be made to the lead contact, Nicholas Tatonetti (Nicholas.Tatonetti@cshs.org).

#### Materials availability

This study did not generate new unique materials.

#### Data and code availability

Patient clinical information cannot be shared due to our data use agreement. Summaries of the patient cohorts are provided in the main and Supplementary tables and are censored at a count of 10. The results of analyses are provided in the main and Supplementary tables.

All scripts used for data preparation and analysis are available from Zenodo as Jupyter Notebooks (https://doi.org/10.5281/zenodo.10034023).

### Ethics statement

The study is approved by the CUIMC Institutional Review Board no. AAAL0601 and the requirement for informed consent was waived. A data request associated with this protocol was submitted to the Tri-Institutional Request Assessment Committee of New York-Presbyterian/Columbia and Cornell and approved.

### Software

We used MySQL 5.7.35 and Python 3.9.10 with the lifelines 0.25.10, numpy 1.19.5, pandas 1.2.3, pymysql 1.0.2, pickle, scipy 1.6.2, and sklearn 0.24.2 libraries in this study.

### Preparation for data modeling and statistical modeling

For each visit, we identified the age of the patient at the start of the visit as (i) birth to 13 years old, (ii) 13–19 years old, (iii) 19–60 years old, and (iv) over 60 years old and if the patients indicated their sex as female. For each visit, we identified whether the patient indicated their race(s) as (i) American Indian or Alaskan Native, (ii) Asian, (iii) Black or African American, (iv) Native Hawaiian or Other Pacific Islander, or (v) White and whether the patient indicated their ethnicity as of Hispanic or Latino or Spanish Origin. All demographic variables were treated as a binary categorical variables with 1 indicating that the patient was a part of the age group, or self-identified as female, or self-identified as the specific race or ethnicity, or had a diagnosis code listed during that visit and 0 indicating the inverse. Additionally, we used the start date of the visit to categorize the visit by month between February 2020 and March 2022. In addition to the ICD-10 diagnosis code for COVID-19 (U07.1), we identified 16,220 distinct ICD-10 clinical diagnosis codes listed for patients in the 26-month period, which we generalized to 1,600 distinct category levels codes.

### Training and evaluating the random Forest classifier

A random Forest classifier is an ensemble model consisting of decision tree estimators that individually train on different, randomly selected subsets of the overall training data. It is commonly used in clinical research, as it typically generalizes well on unseen data due to its built-in regularization. We refined the default model by tuning maximum depth and the number of estimators to maximize the AUROC in the independent evaluation set.

After applying the optimized model to the full dataset, we used isotonic calibration to better align the modeled probabilities predicted by the random Forest classifier to the true probability of COVID-19 diagnosis. Isotonic calibration is a non-parametric method that fits a monotonically increasing function to the predicted probabilities and adjusts them accordingly.

### Identifying phenotypes associated with COVID-19

Clinical diagnosis data from each visit between February 2020 and March 2022 were mapped from the ICD-10 vocabulary to PheCodes. Additionally, historical condition data from our clinical data warehouse were mapped from SNOMED vocabulary to PheCodes. For visits with a follow-up within each time interval (e.g., within 1 week), we discerned the visits where the PheCode was observed in the follow-up and the visits where the PheCode was not observed and compared between the distributions using a MWU test. In the manuscript and tables, p values of 0 are presented as p < 2.225E−308 (the minimum value for a float object in Python), while p values of 0 are recast as half the minimum non-zero p value per test for stylistic purposes in figures. In evaluating instances where the patient was not previously diagnosed with the condition, we eliminated all patients who had a previous history of the condition (i.e., had the diagnosis prior to the start of the visit).

### Cox proportional hazards modeling and Kaplan-Meier curve fitting

We used a Cox proportional hazards model to determine and statistically evaluate the HRs associated with COVID-19 probability. COVID-19 probability consisted of the risk score as output by the previously trained random Forest model. We adjusted for covariates as described in the main text. From our cases visits (those visits where the patient returned with the condition within 1 year), we identified the time to event as the time from the end of the preceding visit to the first instance of the condition within one year of the visit. In our non-case visits, we censored the data at the final interaction with NYP/CUIMC within the time period. To build Kaplan-Meier curves, we stratified our data by the COVID-19 probability of the preceding visit (≤0.2, >0.2, and ≤0.4; >0.4 and ≤0.6; >0.6 and ≤0.8; and >0.8) and fit individual curves to each stratified dataset.

To adjust for the confounding effect of strongly correlated phenotypes when conducting the multivariate Cox proportional hazards analysis, we removed the 30 variables that were determined to have the strongest association with COVID-19 diagnosis. To do so, we adapted the high dimensional propensity score method to calculate the Bross bias multiplier (BB) score and relative risk (RR) score for each clinical condition variable. The BB score quantifies the possible magnitude of bias from a particular variable, calculated from the prevalence of the variable and the RR univariate association between the variable and outcome. We filtered for variables that had a BB score of more than 1.5, at least 10 patients, and selected the 30 variables with the highest BB scores.

## Supplemental information

Document S1. Figures S1 and S2 and Table S1

Table S2. Frequency of patients visits start dates (by month) used for model training, model evaluation and all visits between February 2020 and March 2022

Table S3. Frequency of ICD-10 diagnoses in patients visits used for model training, model evaluation and all visits between February 2020 and March 2022

Table S4. Importance for all features in the random forest classifier with non-zero importance and Wasserstein distance between distribution where the feature is observed and the feature is not observed Note: negative Wasserstein distance indicates that the average COVID-19 probability in the set of visits where the feature was observed is less than the average of the set where the feature was not observed.

Table S5. Mann-Whitney U test results for all conditions tested at each time period

Table S6. Hazard ratios of COVID-19 probability from Cox proportional hazards modeling at each time period

Table S7. Hazard ratios of covariates from Cox proportional Hazards modeling for select conditions within 1 year ± indicates that covariate was not included in the multivariate model due to convergence.

Document S2. Article plus supplemental information
