# Supplementary material for: Using machine learning probabilities to identify effects of COVID-19
Source: Patterns (N Y). 2023 Dec 1;4(12):100889. doi: 10.1016/j.patter.2023.100889 (PMC10724367; doi:10.1016/j.patter.2023.100889)
Supplement: Document S1. Figures S1 and S2 and Table S1 [file mmc1.pdf]

Patterns, Volume *4*

Supplemental information

Using machine learning

probabilities to identify effects of COVID-19

Vijendra Ramlall, Undina Gisladottir, Jenna Kefeli, Yutaro Tanaka, Benjamin May, and Nicholas Tatonetti

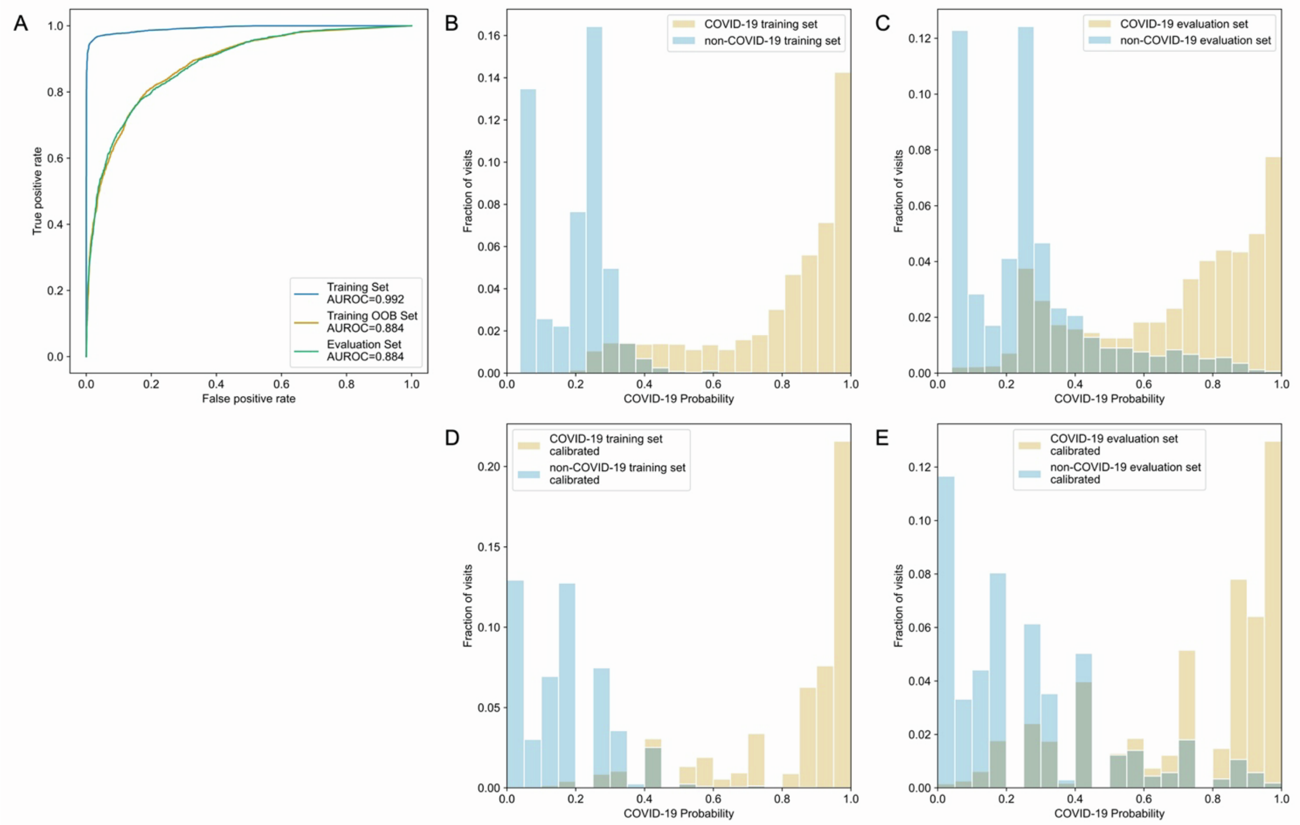

Figure S1 Model performance evaluation (A) ROC curves of training set, training set using out-of-bag estimates and evaluation set in the initial model prior to optimization. Distribution of COVID-19 probabilities outputted by the optimized model in the training (B and D) and evaluation sets (C and E) prior to calibration (B and C) and after calibration (D and E).

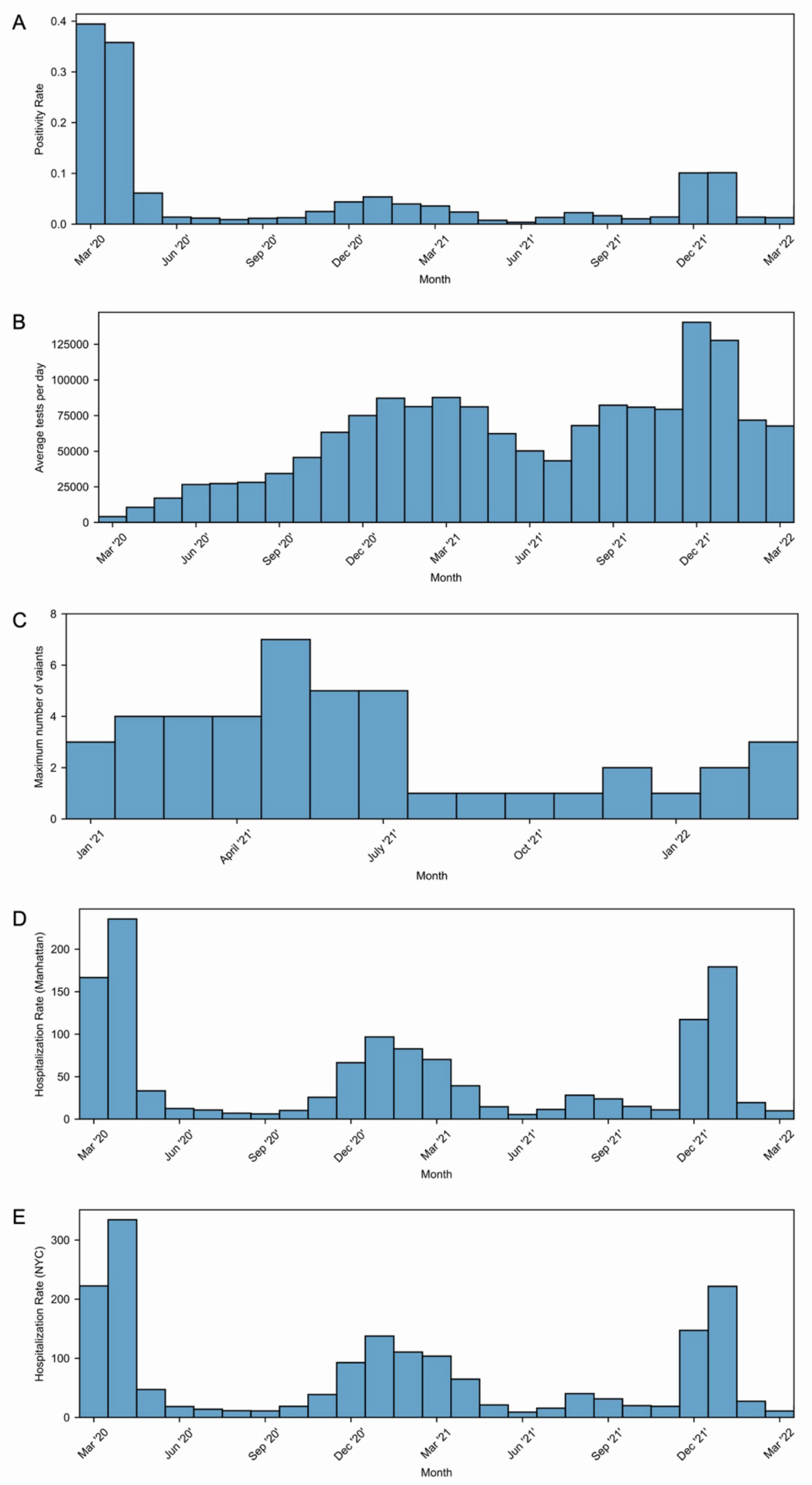

Figure S2 New York City Observations During Study Period Plots show the (A) positivity rate, (B) total number of tests, (C) the number of variants observed, (D) the hospitalization rate in Manhattan and (E) the hospitalization rate in New York City by month during the study period. Data source: https://github.com/nychealth/coronavirus-data/blob/master/trends/hosprate-by-modzcta.csv.

Table S1 Demographics of patients used for model training, model evaluation and all patients between February 2020 and March 2022.

	Model Training Set		Model Evaluation Set		All Visits
	non-COVID-19	COVID-19	non-COVID-19	COVID-19	Feb 2020 - Mar 2022
**N(patients)**	4,606	4,178	4,592	4,137	434,152
**Age Child (< 13) (% of patients)**	355<br>7.71%	194<br>4.64%	315<br>6.86%	189<br>4.57%	49,804<br>11.5%
**Age Adolescent (≥ 13 and < 19) (% of patients)**	140<br>3.04%	89<br>2.13%	157<br>3.42%	93<br>2.25%	18,906<br>4.35%
**Age Adult (≥ 19 and < 60) (% of patients)**	2,320<br>50.4%	1,872<br>44.8%	2,256<br>49.1%	1,817<br>43.9%	212,531<br>49.0%
**Age Senior (≥ 60) (% of patients)**	1,791<br>38.9%	2,023<br>48.4%	1,864<br>40.6%	2,038<br>49.3%	152,911<br>35.2%
**Self-identified Sex as Female (% of patients)**	2,716<br>59.0%	2,179<br>52.2%	2,847<br>62.0%	2,149<br>51.9%	252,309<br>58.1%
**Self-identified as American Indian or Alaskan Native (% of patients)**	14<br>0.304%	16<br>0.383%	11<br>0.240%	< 10<br>< 0.242%	971<br>0.224%
**Self-identified as Asian (% of patients)**	107<br>2.32%	110<br>2.63%	117<br>2.55%	101<br>2.44%	10,667<br>2.46%
**Self-identified as Black or African American (% of patients)**	713<br>15.5%	714<br>17.1%	694<br>15.1%	710<br>17.2%	62,094<br>14.3%
**Self-identified as Native Hawaiian or Other Pacific Islander (% of patients)**	< 10<br>< 0.217%	< 10<br>< 0.239%	< 10<br>< 0.218%	< 10<br>< 0.242%	534<br>0.123%
**Self-identified as White (% of patients)**	1,875<br>40.7%	1,470<br>35.2%	1,946<br>42.4%	1,470<br>35.5%	171,309<br>39.5%
**Self-identified as Hispanic or of Latino or Spanish Origin (% of patients)**	1,414<br>30.7%	1,654<br>39.6%	1,346<br>29.3%	1,586<br>38.3%	126,686<br>29.2%
